# Clinical value of LHPP‐associated microRNAs combined with protein induced by vitamin K deficiency or antagonist‐II in the diagnosis of alpha‐fetoprotein‐negative hepatocellular carcinoma

**DOI:** 10.1002/jcla.23071

**Published:** 2019-11-06

**Authors:** Zeyu Tian, Tanbo Yu, Hongyan Wei, Ning Ning

**Affiliations:** ^1^ Department of Hepatobiliary Surgery The First Affiliated Hospital of Hunan Normal University Changsha China; ^2^ Department of Pharmacy The First Affiliated Hospital of Hunan Normal University Changsha China; ^3^ Department of Medical Administration The First Affiliated Hospital of Hunan Normal University Changsha China

**Keywords:** alpha‐fetoprotein, diagnosis, hepatocellular carcinoma, microRNA, protein induced by vitamin K deficiency or antagonist‐II

## Abstract

**Background:**

Alpha‐fetoprotein (AFP) has received extensive attention in the differential diagnosis of hepatocellular carcinoma (HCC), especially for AFP‐negative HCC (AFP‐NHCC). The current study aimed to explore the value of targeted regulation of LHPP expression‐related microRNAs (miRs) and protein induced by vitamin K deficiency or antagonist‐II (PIVKA‐II) in the differential diagnosis of AFP‐NHCC.

**Methods:**

A retrospective study was conducted on a testing set—including 214 AFP‐NHCC patients, 200 cirrhosis, and 210 controls, and a validation set—including 140 AFP‐NHCC patients, 134 cirrhosis, and 128 controls recruited from The First Affiliated Hospital of Hunan Normal University. Serum miRs were examined using quantitative real‐time PCR method. Serum PIVKA‐II was measured by enzyme‐linked immunosorbent assay.

**Results:**

Compared with adjacent tissues, LHPP protein levels in cancer tissues were significantly decreased (*P* < .05). Predictive software and dual‐luciferase reporter assays showed that miR‐363‐5p and miR‐765 can target LHPP expression. Serum miR‐363‐5p, miR‐765, and PIVKA‐II levels were significantly higher in AFP‐HCC patients than in cirrhosis and controls. A logistic regression model combining miR‐363‐5p, miR‐765, and PIVKA‐II was performed. This model presented a high discriminating value (AUC: 0.930, sensitivity/specificity: 79.4%/95.4%) than any single indicator. In the validation set, this model still showed a high discriminating value (AUC: 0.936, sensitivity/specificity: 83.6%/94.7%).

**Conclusion:**

Current model combining serum miR‐363‐5p, miR‐765, and PIVKA‐II has potential significance for diagnosis of AFP‐NHCC.

## 1 INTRODUCTION

For decades, hepatocellular carcinoma (HCC) screening relied primarily on ultrasound imaging and alpha‐fetoprotein (AFP). Due to technical limitations, ultrasound images are often unrecognizable for HCC nodules, especially less than 1 cm.^1^, ^2^ Unexpectedly, AFP is measured separately in early HCC with a missed diagnosis rate of 40%.^3^ AFP‐negative hepatocellular carcinoma (AFP‐NHCC) is an important type of HCC that currently causes many patients to lose early diagnosis and treatment, especially in patients with tumors less than 3 cm.^4^ The clinical symptoms of AFP‐NHCC patients are usually mild and lack specificity, and their clinical diagnosis relies mainly on other tumor markers or imaging. Protein induced by vitamin K deficiency or antagonist‐II (PIVKA‐II) is believed to be a suitable biomarker specific for HCC.^5^ However, the sensitivity of PIVKA‐II is still not satisfactory.^6^ In addition, due to the small size of the AFP‐NHCC tumor, imaging examination is prone to miss. It is reported that the diagnostic rates of AFP‐NHCC patients by CT, MRI, and B ultrasound are about 50.9%, 50.0%, and 10.4%, respectively.^7^ In addition, liver nodular lesions such as cirrhosis regenerative nodules, hepatic focal nodular hyperplasia, hepatic adenomas may also have HCC‐like imaging findings, making AFP‐NHCC easily misdiagnosed as benign disease, and thus lost the opportunity for early treatment.^2^, ^8^

In 2018, Hindupur et al^9^ discovered a new HCC suppressor protein‐LHPP, in the mouse HCC model, and they also revealed its potential anticancer mechanism. They found that (a) with the development of HCC tumors, the expression of LHPP protein in mouse HCC cancer tissues gradually decreased, while the level of LHPP in the adjacent tissues was normal; (b) The Cancer Genome Atlas (TCGA) data showed that the severity of HCC and the life expectancy of patients were significantly correlated with the level of LHPP in the tissues, and the patients with low LHPP expression had a lower median survival time than those with high LHPP expression for nearly 2 years; (c) when the expression of LHPP in cells is downregulated, the level of proteomic phosphorylation in the cells is significantly increased, thereby causing uncontrolled cancer cell proliferation. The above study suggests that the decreased expression of LHPP in tissues is an important factor in promoting the formation of HCC. However, since the above experiments are based on the protein quantification of LHPP in cancer tissues, it is not conducive to the spread of cancer screening. Therefore, searching for non‐invasive markers involved in the regulation of LHPP expression is the focus of our study.

MicroRNA (miR) plays an important role in many biological processes.^10^, ^11^, ^12^, ^13^ In current study, firstly, we identified the miRs that are targeted for downregulating LHPP expression through bioinformatics software and luciferase reporter gene assay. Then, we evaluated the significance of miRs and PIVKA‐Ⅱ in distinguishing AFP‐NHCC. In addition, logistic regression model was built for AFP‐NHCC prediction.

## 2 MATERIALS AND METHODS

### 2.1 Ethics approval and consent to participate

This study was approved by the Ethics Committee of The First Affiliated Hospital of Hunan Normal University (L20180104). Written informed consent was provided in accordance with the Declaration of Helsinki.

### 2.2 Testing set

We recruited 214 patients AFP‐NHCC between April 2016 and January 2018 at The First Affiliated Hospital of Hunan Normal University, Changsha, China. AFP‐NHCC was confirmed by liver puncture or histopathology examination. Two hundred patients with cirrhosis and two hundred and ten controls were also recruited.

### 2.3 Logistic regression model establishment

A regression formula for AFP‐NHCC prediction was established. The formula is as follows: Logit (P) = X_0_ + X_1_Y_1_ + X_2_Y_2_ + X_3_Y_3_+…+X_n_Y_n_ = ln[p/(1‐p)], “p” means the incident probability (AFP‐NHCC), “n” means the number of interference factor, “X” means the influence coefficient of each interference factor, and “Y” means the value of each interference factor.

### 2.4 Validation set

One validation set from The First Affiliated Hospital of Hunan Normal University (Changsha, China) was used to assess the above logistic regression model including a total of 140 AFP‐NHCC, 134 cirrhosis, and 128 controls between February 2018 and April 2019.

### 2.5 Serum and tissue specimens

Peripheral blood was collected from AFP‐NHCC and cirrhosis before receiving treatment and healthy controls at the time of admission to the Medical Examination Center. In addition, eight pairs of cancer and adjacent tissues (>3 cm from the edge of cancer tissue) from AFP‐NHCC patients who underwent surgical treatment were enrolled.

### 2.6 Cell culture and cell transfection

Human HCC cell line Hep G2 (Institute of Biosciences Cell Resource Center, Chinese Academy of Sciences, Shanghai, Lot number: ZQ0022) and normal liver cell line LO2 (Institute of Biosciences Cell Resource Center, Chinese Academy of Sciences, Shanghai, Lot number: ZQ0013) were cultured in RPMI‐1640 (Hyclone, Lot number: SH30809.01) medium supplemented with 10% fetal bovine serum. The culture conditions were 37°C, and the culture was carried out at a saturated humidity of 5% CO_2_. Small interference RNAs (siRNAs) targeting LHPP (si‐LHPP) was obtained from GenePharma Co. ltd (Lot number: W‐19‐09602). The transfection group was divided into two groups, including control group and LHPP inhibitor (si‐LHPP) group. Cell lines were seeded in a six‐well plate, and when the cell confluence reached about 50%, the transfected cell line was immediately mediated with **Lipofectamine 2000** (Invitrogen, Thermo, New York, USA, Lot number: 11668‐027), and the medium was changed 6 h after transfection. After si‐LHPP treatment for 24 hours, the cell lysates were immunoblotted with antibodies against LHPP.

### 2.7 Quantitative Real‐Time PCR (qRT‐PCR)

Cell and serum total RNAs were extracted using Trizol (Invitrogen, Lot number: 15596026). The quality of extracted RNAs was tested by Nanodrop ND 8000 (Invitrogen). RNAs were reverse‐transcribed using PrimeScript™ RT reagent Kit (Takara, Lot number: RR047A). The reverse transcription conditions are set as follows: 42°C (2 minutes), then 37°C (15 minutes), and 85°C (5 seconds). Level of miR was tested by qRT‐PCR using SYBR‐Green I Premix EXTaq (Takara, Lot number: DRR036A). U6 was used as the endogenous control. The primers sequences, which were synthesized by Beijing Tianyi Huiyuan Bioscience & Technology Inc, were as follows: miR‐765 (forward: 5′‐CGGCTCGGATCCGTTAG‐3′ and reverse: 5′‐CGACTACCGTTAGCTAGA‐3′); miR‐363‐5p (forward: 5′‐CCGTATTACGCTAGTCAGCAG‐3′ and reverse: 5′‐GGCACCAGTACTAGACA‐3′); U6 (forward: 5′‐CGCTTCGGCAGGCATTATATAC‐3′ and reverse: 5′‐AAGGGGCCATGCTAATCTT‐3′). The amplification condition is set as follows: 95°C (5 minutes), followed by 45 cycles of 95°C (30 seconds), 60°C (30 seconds), and 72°C (30 seconds). The specificity of the amplification products was analyzed by melting curve. The relative level was calculated by 2^−△Ct^. All reactions were repeated three times.

### 2.8 Serum PIVKA‐II assay

PIVKA‐II was measured by enzyme‐linked immunosorbent assay. The kit was provided by Wuhan Boster Bioengineering Co., Ltd (Lot number: 233887), and the detection process was carried out in strict accordance with the operation instructions. The procedure was as follows: All serum samples were sequentially added to the microplates and incubated with the antibody for 40 minutes at room temperature (adding PIVKA‐II standards at a concentration of 100 mAU/mL, 50 mAU/mL, 10 mAU/mL, and positive and negative controls), washing the plate five times. The enzyme‐labeled monoclonal antibody was incubated at room temperature for 40 minutes in the dark, washed for five times, and the substrate was reacted for 15 minutes to terminate the reaction. The absorbance (A) was measured using an American Thermo Fisher Scientific Nanodrop ND2000. The kit performance includes the following: (a) accuracy—the linear regression of the standard and the expected concentration correlation coefficient R value is greater than or equal to 0.9900; (b) sensitivity—the lowest detection concentration is less than 1.0 mAU/mL; (c) specificity—does not cross‐react with other soluble structural analogs; and (d) repeatability—the coefficient of variation between the plate was <15%. In this study, we calculated the intra‐ and inter‐assay coefficients of variation for serum PIVKA‐II assays to assess the repeatability and precision of the experiments.

### 2.9 Western blot analysis

Total protein was extracted using RIPA buffer (Beyotime, Lot number: P0013B) containing protease inhibitors, and protein levels were detected using the BCA reagent (Beyotime, Lot number: P0012). A protein sample having a loading of 30 μg per well was separated by sodium dodecyl sulfate‐polyacrylamide gel electrophoresis (SDS‐PAGE) and then electrotransferred to a nitrocellulose (NC) membrane. After blocking with 5% skim milk for 1 hour at room temperature, membrane was incubated overnight with LHPP (rabbit monoclonal, 1:2000; Cell Signaling Technology, Lot number: XY15759‐1) and β‐actin (rabbit monoclonal, 1:3000; Cell Signaling Technology, Lot number: YY‐71603) primary antibody at 4°C. Then, the membrane was incubated with a secondary anti‐rabbit antibody (1:4000; Cell Signaling Technology, Lot number: BS10044) for 1 hour. Finally, the membrane was visualized by ECL‐PLU (Amersham Biosciences, Lot number: EWC101).

### 2.10 Immunohistochemical staining

All tissue samples were fixed with 4% formaldehyde, and then, the samples were dehydrated and sectioned. The sections were blocked at room temperature for 1 hour (5% serum), and the monoclonal antibody LHPP (rabbit monoclonal, 1:2000; Cell Signaling Technology, Lot number: XY15759‐1) was added, and the alkaline phosphatase secondary antibody (rabbit monoclonal, 1:1000; Cell Signaling Technology, Lot number: XY‐37831) was incubated at 4°C. The streptavidin‐peroxidase and diaminobenzidine thermostat color‐blocking sheets were separately added. The results of immunohistochemistry were analyzed by ImagePro Plus, and the percentage of positive cells and the staining intensity of positive cells were scored. The formula was X × Y. X represents the percentage of positive cells: x = 0, no positive cells; x = 1, positive cells are 1%‐10%; x = 2, positive cells are 11%‐50%; x = 3, positive cells are 51%‐80%; and x = 4 positive cells account for more than 81%. Y represents positive cell staining intensity: y = 0, negative; y = 1, weakly positive; y = 2, moderately positive; y = 3, strong positive.

### 2.11 Luciferase reporter gene assay

Artificially synthesized miR‐363‐5p (5′‐UUAAUCACUUGAUACUGA‐3′), miR‐765 (5′‐ACTGCUUUACUUCGATAGAA‐3′), miR‐632 (5′‐UAAAUUUCACACUAAUACU‐3′), miR‐30b‐3p (5′‐ACTCUCCCAAUUACAGAGG‐3′), and miR‐644a (5′‐AAACUUCACUCAauGAGU‐3′) mimics and LHPP 3′UTR (upstream: 5′‐GCCATTAGCTAGACGGTA‐3′; downstream: 5′‐GGCTCCGATCTAGACT‐3′) were transferred to the pmiR‐RB‐Report™ reporter gene by restriction enzymes SpeI and Hind III (Beijing Huaketai Biotechnology Co., Ltd.). Wide‐type (WT) LHPP 3′UTR‐WT luciferase reporters and mutant type (Mu)‐LHPP 3′UTR‐Mu luciferase reporters were co‐transfected with pRL‐SV40 (Invitrogen, Lot number: 1442953) and corresponding miR‐mimic/miR‐control into Hep G2 cells, and luciferase activity was determined 48 hours later.

### 2.12 Statistical analysis

SPSS 19.0 was used. Differences among normally distributed data were evaluated by *t* test or ANOVA; otherwise, Mann‐Whitney *U* test and Kruskal‐Wallis *H* test were used. *P* < .05 was considered significant.

## 3 RESULTS

### 3.1 Expression of LHPP protein in AFP‐NHCC patients and prediction and validation of miRs regulating LHPP expression

**Figure 1:**
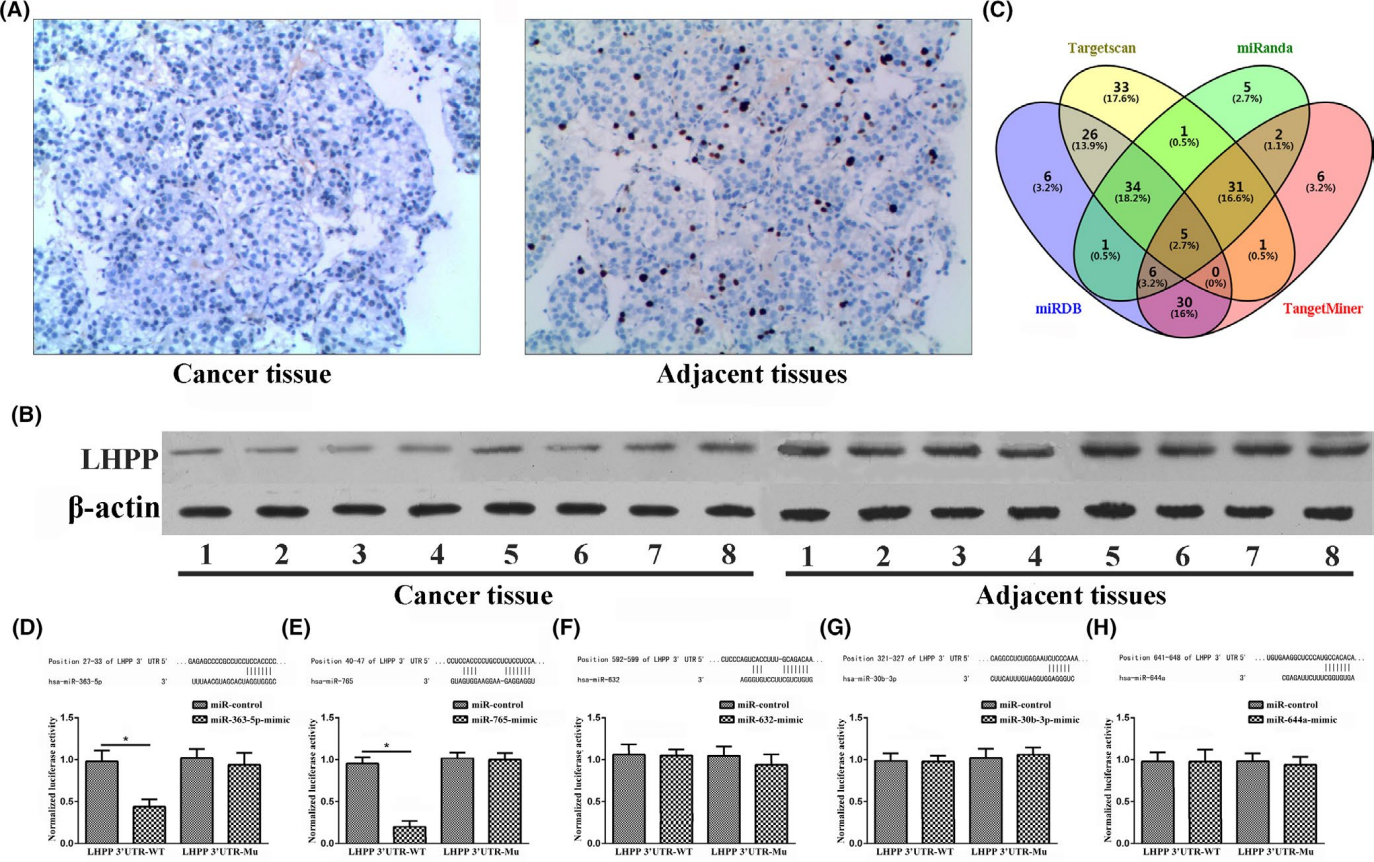
LHPP protein expression levels in AFP‐NHCC patients and prediction and validation of miRs regulating LHPP expression. A, Detection of protein levels in tissues of AFP‐NHCC patients by immunohistochemistry; (B) detection of protein levels in tissues of AFP‐NHCC patients by Western blot; (C) Targetscan, miRanda, miRDB, and TangetMiner software to predict the miRs targeting LHPP; (D‐H): luciferase reporter gene assay: (D) miR‐363‐5p; (E): miR‐765; (F): miR‐632; (G): miR‐30b‐3p; (H): miR‐644a

The results of immunohistochemical staining are shown in Figure 1A. Compared with adjacent tissues (21.2 ± 2.1), the staining intensity of LHPP protein in cancer tissues (4.8 ± 0.9) decreased significantly (*P* < .05). The immunohistochemical result of using PBS instead of primary antibody as a negative control is shown in Figure S1. Results of Western blot analysis of 8 pairs of AFP‐NHCC patients are shown in Figure 1B. Compared with adjacent tissues (5.2 ± 0.2), LHPP protein levels in cancer tissues (1.2 ± 0.1) were significantly decreased (*P* < .05). The validation of the anti‐LHPP antibody is shown in Figure S2. Our results showed that the anti‐LHPP antibody does not have cross‐reaction with other proteins.

Using Targetscan, miRanda, miRDB, and TangetMiner software to predict the miRs targeting LHPP, a total of 5 miRs (miR‐363‐5p, miR‐765, miR‐632, miR‐30b‐3p, and miR‐644a) were simultaneously predicted which may be involved in the targeted regulation of LHPP, Figure 1C. The luciferase reporter gene assay showed that the luciferase activity of the miR‐363‐5p mimic group (0.43 ± 0.07) and the miR‐765 mimic group (0.21 ± 0.03) was significantly lower than that of the negative control group (0.95 ± 0.08, 0.93 ± 0.05) in the wild‐type LHPP (*P* < .05), but there was no significant difference in the mutant LHPP (*P* > .05), Figure 1D‐H.

### 3.2 The relationship between serum miR‐363‐5p, miR‐765, and PIVKA‐II levels and clinical features of AFP‐NHCC and their differential diagnosis value for AFP‐NHCC

**Figure 2:**
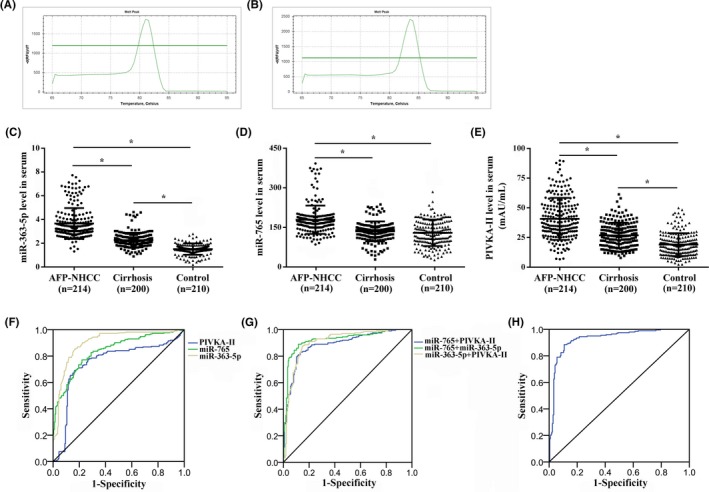
Relationship between serum miR‐363‐5p, miR‐765, and PIVKA‐II levels and clinical value in AFP‐NHCC patients. A, The melting peak of miR‐363‐5p. B, The melting peak of miR‐765. C, miR‐363‐5p level in serum. D, miR‐765 level in serum. E, PIVKA‐II level in serum. F, Differential diagnosis value of single index for AFP‐NHCC. G, Differential diagnosis value of two indicators for AFP‐NHCC. H, Differential diagnosis value of three indicators for AFP‐NHCC. ^*^ *P* < .05

The main baseline characteristics of the studied subjects are illustrated in Table 1. No significant difference was observed (*P* > .05). The melting peaks of miR‐363‐5p and miR‐765 were single, indicating that the primers did not form primer dimers and there was no non‐specific amplification (Figure 2A,B).

**Table 1:** Comparison of baseline characteristics between AFP‐NHCC, cirrhosis, and healthy people (testing set)

Characteristics	AFP‐NHCC (n = 214)	Cirrhosis (n = 200)	Controls (n = 210)	*P*
Age (y), median (IQR)	53 (44, 67)	51 (42, 65)	53 (45, 67)	.693^a^
Male sex (n), %	168 (78.50%)	154 (77.00%)	162 (77.14%)	.920^b^
Smoking (n), %	128 (59.81%)	112 (56.00%)	121 (57.62%)	.732^b^
Drinking (n), %	145 (67.76%)	126 (63.00%)	134 (63.81%)	.551^b^
AFP (µg/L), mean ± SD	10.8 ± 3.1	11.1 ± 3.5	10.7 ± 2.7	.098^c^

a Kruskal‐Wallis *H* test.

b Chi‐square test.

c ANOVA test.

The intra‐assay CV value of serum PIVKA‐II was 7.4%, and the inter‐assay CV value was 8.9%, which was less than the 15% specified in the kit, suggesting that the test results have good repeatability and precision. Serum miR‐363‐5p and PIVKA‐II levels were significantly higher in AFP‐HCC (miR‐363‐5p: 3.7 ± 1.0; PIVKA‐II: 42.0 ± 9.2 mAU/mL) patients than in cirrhosis (miR‐363‐5p: 2.3 ± 0.7; PIVKA‐II: 27.4 ± 5.7 mAU/mL) and controls (miR‐363‐5p: 1.7 ± 0.4; PIVKA‐II: 15.8 ± 4.6 mAU/mL), while serum miR‐363‐5p and PIVKA‐II levels were significantly higher in patients with cirrhosis than in controls (*P* < .05, Figure 2C,E). Serum miR‐765 was significantly increased in patients with AFP‐HCC (183.1 ± 22.6) compared with cirrhosis (144.0 ± 18.9) and controls (142.9 ± 19.6, *P* < .05, Figure 2D).

To estimate the diagnostic value of miR‐363‐5p, miR‐765 and PIVKA‐II in AFP‐NHCC, ROC was constructed using the following model: AFP‐NHCC *vs*. non‐AFP‐NHCC (controls + cirrhosis), Figure 2F‐H and Table 2. We found that the combination of the three indicators possessed a higher specificity (95.4%) for differentiating AFP‐NHCC from non‐AFP‐NHCC.

**Table 2:** Comparisons of the AUC of miR‐765, miR‐363‐5p, and PIVKA‐II in the subgroups

Group	AUC	95% CI	*P*	Se (%)	Sp (%)
PIVKA‐II	0.749	0.698‐0.800	<.001	65.4	84.6
miR‐363‐5p	0.901	0.870‐0.933	<.001	78.5	87.3
miR‐765	0.838	0.800‐0.876	<.001	77.6	78.0
PIVKA‐II + miR‐765	0.887	0.854‐0.920	<.001	79.0	91.0
PIVKA‐II + miR‐363‐5p	0.906	0.876‐0.936	<.001	87.4	82.7
miR‐765 + miR‐363‐5p	0.923	0.895‐0.952	<.001	88.8	87.8
PIVKA‐II + miR‐765 + miR‐363‐5p	0.930	0.904‐0.956	<.001	79.4	95.4

Abbreviations: AUC, area under the receiver operating characteristic curves; CI, confidence interval; Se, sensitivity; Sp, specificity.

**Figure 3:**
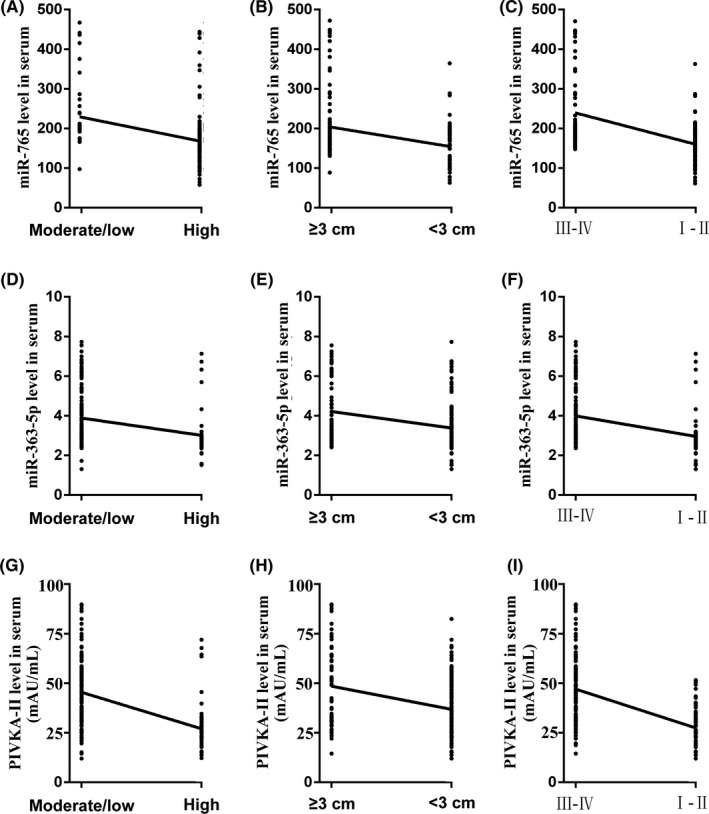
Correlation of miR‐363‐5p, miR‐765, and PIVKA‐II levels in relation to clinical parameters of the AFP‐NHCC cases. A, Correlation between miR‐765 and differentiation. B, Correlation between miR‐765 and tumor size. C, Correlation between miR‐765 and TNM stage. D, Correlation between miR‐363‐5p and differentiation. E, Correlation between miR‐363‐5p and tumor size. F, Correlation between miR‐363‐5p and TNM stage. G, Correlation between PIVKA‐II and differentiation. H, Correlation between PIVKA‐II and tumor size. I, Correlation between PIVKA‐II and TNM stage

In addition, we detected the correlation between miR‐363‐5p, miR‐765, and PIVKA‐II levels and clinical parameters. As shown in Figure 3 and Table 3, miR‐363‐5p, miR‐765, and PIVKA‐II were significantly correlated with differentiation, tumor size, and TNM stage.

**Table 3:** Correlation analysis of miR‐765, miR‐363‐5p, and PIVKA‐II in relation to clinical parameters of AFP‐NHCC

Characteristics	miR‐765		miR‐363‐5p		PIVKA‐II	
	*r*_Spearman_	*P*	*r*_Spearman_	*P*	*r*_Spearman_	*P*
Differentiation (high vs moderate/low)	−0.362	<.001	−0294	<.001	−0.456	<.001
Tumor size (<3 vs ≥3 cm)	−0.325	<.001	−0.303	<.001	−0.331	<.001
TNM stage (I‐II vs III‐IV)	−0.518	<.001	−0.372	<.001	−0.506	<.001

### 3.3 The logistic regression model for AFP‐NHCC

MiR‐363‐5p, miR‐765, and PIVKA‐II were included in the logistic regression model. The final model for AFP‐NHCC prediction was as follows: Logit (P) = 4.382 + 0.507(miR‐363‐5p)‐0.023(miR‐765)‐0.068(PIVKA‐II), the identification value of this model was high with AUC of 0.930 (Figure 2H), and the probability was 0.407, which means if the probability was <0.407, it was classified into the AFP‐NHCC; on the contrary, it was classified into non‐AFP‐NHCC.

### 3.4 Validation of the logistic regression model

The validity of the logistic regression model was assessed in one external validation set from our hospital. A total of 140 AFP‐NHCC, 134 cirrhosis, and 128 controls were recruited. The main baseline characteristics of the studied subjects are illustrated in Table 4. No significant difference was observed in baseline characteristics (*P* > .05).

**Table 4:** Comparison of baseline characteristics between AFP‐NHCC, cirrhosis, and healthy people (validation set)

Characteristics	AFP‐NHCC (n = 140)	Cirrhosis (n = 134)	Controls (n = 128)	*P*
Age (y), median (IQR)	51 (43, 68)	52 (41, 66)	55 (43, 64)	.437^a^
Male sex (n), %	96 (68.57%)	87 (64.93%)	86 (67.19%)	.812^b^
Smoking (n), %	73 (52.14%)	67 (48.91%)	69 (53.91%)	.818^b^
Drinking (n), %	83 (59.29%)	77 (57.46%)	76 (59.38%)	.938^b^
AFP (µg/L), mean ± SD	10.9 ± 3.5	10.6 ± 3.0	10.8 ± 3.7	.322^c^

a Kruskal‐Wallis *H* test.

b Chi‐square test.

c ANOVA test.

By using the formula, the probabilities of 117 (out of 140) AFP‐NHCC patients were lower than 0.407, and the probabilities of 248 (out of 262) healthy controls were more than 0.407 in the validation set. The sensitivity/specificity of the model for AFP‐NHCC was 83.6%/94.7%, with the AUC of 0.936.

## 4 DISCUSSION

Although there are great developments in the current treatment of HCC, including surgical resection, liver transplantation, adjuvant therapy, and interventional therapy, many HCC patients are diagnosed after the occurrence of relevant clinical symptoms.^14^, ^15^, ^16^ Therefore, identification of an effective diagnostic model for HCC is of great importance for patients, particularly for AFP‐NHCC patients. Recently, LHPP is reported to play an essential role in inhibiting human HCC progression by regulating phosphatidylinositol‐3‐kinase/protein kinase B (PI3K/AKT) signaling pathway, and the loss of LHPP expression is also associated with reduced survival in HCC.^9^ Zheng et al^17^ also found that LHPP expression levels were markedly reduced in human cervical cancer tissue samples compared to the adjacent normal tissue. In addition, over‐expressing LHPP suppressed cervical cancer cell proliferation and metastasis. Hence, we hypothesized that LHPP might be also involved in the development of AFP‐NHCC and the study of LHPP is conducive to the early diagnosis of AFP‐NHCC. However, since the above experiments are based on protein quantification of LHPP in cancer tissues, it is not conducive to the spread of cancer screening. MiRs are a class of non‐coding RNAs that affect tumor progression through a variety of epigenetic regulatory pathways. Therefore, looking for miRs involved in the regulation of LHPP expression is the focus of our study.

Recent studies have found that many miRs are involved in the development of tumors, and their tissue and serological levels can be used as diagnostic markers for tumors. MiR can be used as a tumor marker based on the following: (a) It is stable in blood and tissues, and the detection method is relatively convenient and convenient and meets the conditions as a tumor marker^18^; (b) it has the stage specificity of tumorigenesis, and the same tumor has different miR expression profiles in different stages of tumor development^19^; and (c) it participates in all stages of tumorigenesis, development, and metastasis.^20^ Comparing the expression levels of tumor cells with normal cells, the miR expression profiles of the two were significantly different and could be released into the peripheral blood circulation and detected differences.

In our study, the results here indicated that LHPP was markedly reduced in AFP‐NHCC cancer tissues, consistent with previous studies by Hindupur et al.^9^ Targetscan, miRanda, miRDB, and TangetMiner software combined with luciferase reporter detection indicated that miR‐363‐5p and miR‐765 were involved in the targeted regulation of LHPP. Further, the current study screened miR‐363‐5p and miR‐765 to downregulate the expression of LHPP. MiR‐363‐5p and miR‐765 have been found to play essential roles in cancer‐promoting genes in clinical and basic research of HCC. Zhang et al^21^ analyzed the prognosis of 377 patients with HCC, indicating that patients with low‐miR‐363‐5p‐expressing had a better prognosis than those with high serum miR‐363‐5p expression. Xie et al^22^ found that miR‐765 was significantly upregulated in various HCC cell lines and cancer tissues compared with human normal liver cell lines and adjacent tissues, and liposome transfection of miR‐765 mimics to HCC Cell lines can significantly promote the proliferation and tumorigenicity of cancer cells, while downregulating miR‐765 can reverse its cancer‐promoting effect on cells. This study found that serum levels of miR‐363‐5p and miR‐765 in patients with AFP‐NHCC were significantly higher than those in cirrhosis and controls and were related to differentiation, tumor size, and TNM stage, confirming that they are oncogenes in HCC. In recent years, PIVKA‐II is considered to be a novel serological marker for HCC.^6^, ^23^ Our results indicated that PIVKA‐II was significantly higher in AFP‐NHCC patients and were useful for distinguishing AFP‐NHCC from cirrhosis and controls, and the sensitivity was 65.4%, the specificity was 84.6%, which was consistent with previous research results.^23^ In our research, miR‐363‐5p was the most effective indicator (AUC = 0.901) for the diagnosis of AFP‐NHCC than miR‐765 (AUC = 0.838) and PIVKA‐II (AUC = 0.749), but its sensitivity (78.5%) was unsatisfactory. Hence, it might be better to combine multiple hematological parameters to detect AFP‐NHCC.

In the current research, a logistic regression model was established which includes miR‐363‐5p, miR‐765, and PIVKA‐II. It presented a high discriminating value (AUC: 0.930, sensitivity/specificity: 79.4%/95.4%) than any single indicator. Moreover, we validated this model in another validation set. Current model still showed a high discriminating value (AUC: 0.936, sensitivity/specificity: 83.6%/94.7%). In conclusion, current logistic regression model combining serum miR‐363‐5p, miR‐765, and PIVKA‐II has potential significance for the non‐invasive differential diagnosis for AFP‐NHCC.

## CONFLICT OF INTEREST

The authors declare that there is no conflict of interest.

## AUTHOR CONTRIBUTIONS

NN researched literature and conceived the study. ZT and HW were involved in protocol development, gaining ethical approval, patient recruitment, and data analysis. ZT and TY wrote the first draft of the article. All authors reviewed and edited the article and approved the final version of the article.

## ETHICAL APPROVAL

The ethics committee of The First Affiliated Hospital of Hunan Normal University approved this study (L20180104).
