# Supplementary figures and images for: Clinical value of LHPP‐associated microRNAs combined with protein induced by vitamin K deficiency or antagonist‐II in the diagnosis of alpha‐fetoprotein‐negative hepatocellular carcinoma
Source: J Clin Lab Anal. 2019 Nov 6;34(2):e23071. doi: 10.1002/jcla.23071 (PMC7031545; doi:10.1002/jcla.23071)

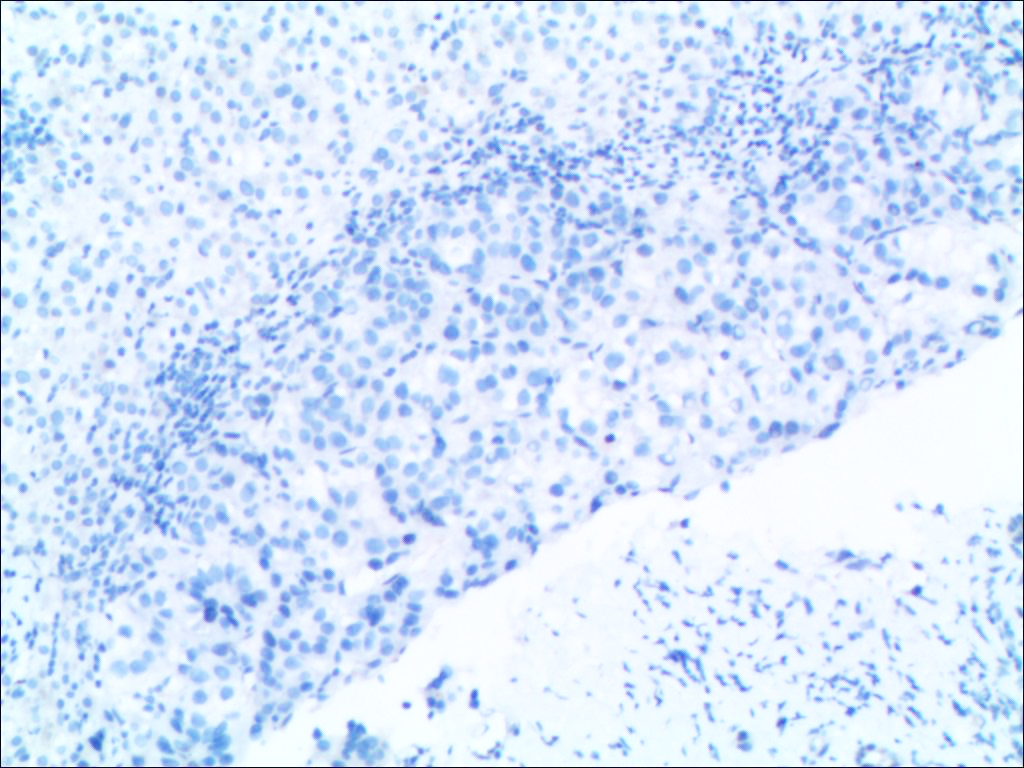

Supplement: Supplementary file 1 [file JCLA-34-e23071-s001.tif]

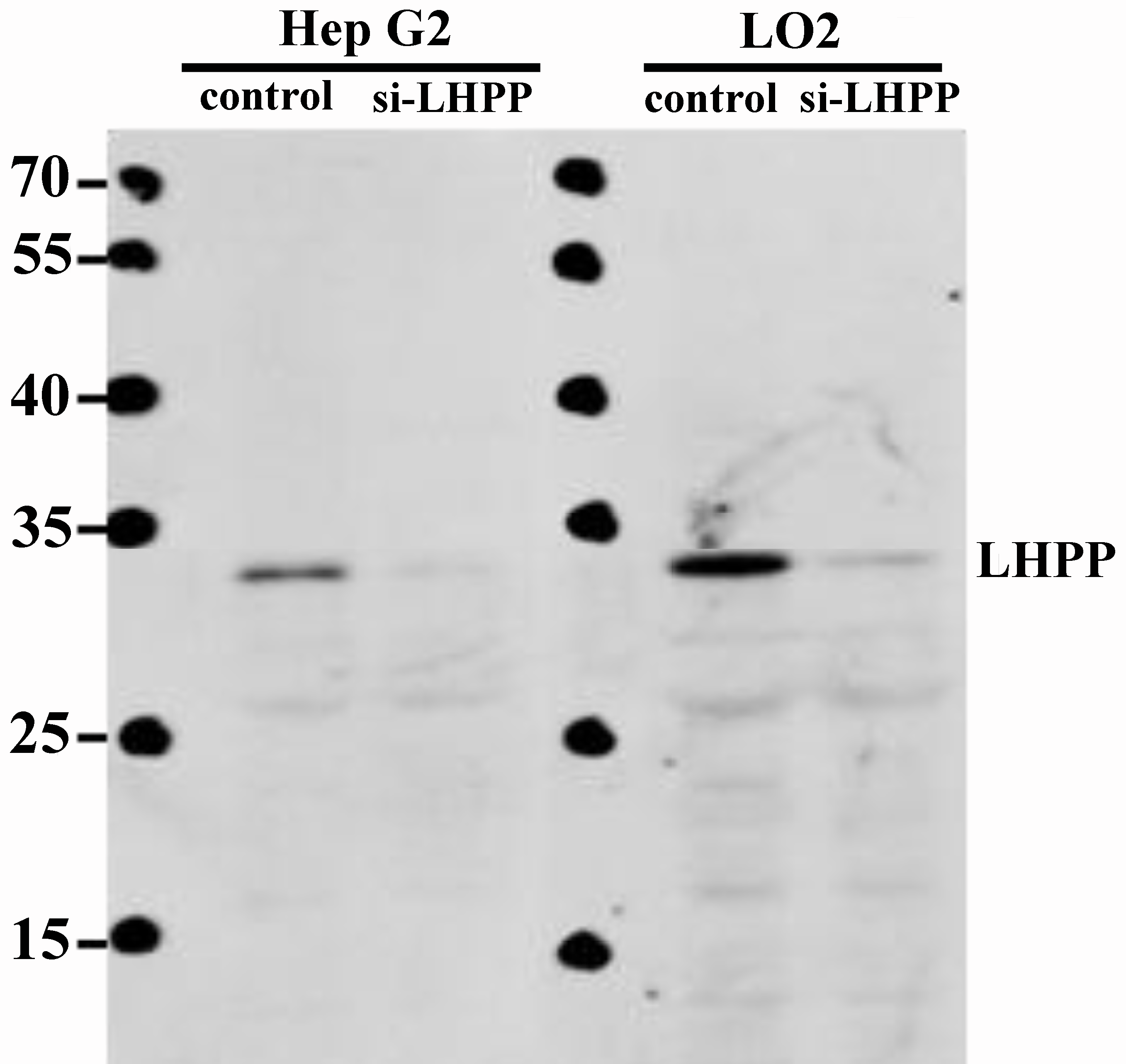

Supplement: Supplementary file 2 [file JCLA-34-e23071-s002.tif]
